# Commentary: SWS Brain-Wave Music May Improve the Quality of Sleep: An EEG Study

**DOI:** 10.3389/fnins.2021.609169

**Published:** 2021-02-01

**Authors:** Jennifer M. Johnson, Simon J. Durrant

**Affiliations:** ^1^School of Health and Social Care, University of Lincoln, Lincoln, United Kingdom; ^2^Lincoln Sleep Research Centre, University of Lincoln, Lincoln, United Kingdom; ^3^School of Psychology, University of Lincoln, Lincoln, United Kingdom

**Keywords:** slow-wave sleep, delta power, music, auditory cognitive neuroscience, sleep

Sleep is important for maintaining health and general well-being. Improving sleep is becoming more important due to the growing prevalence of sleep disorders, with non-pharmacological sleep interventions increasing in popularity (de Niet et al., 2009; Ngo et al., 2013b). When comparing interventions, music-based were the most successful for improving subjective sleep quality (de Niet et al., 2009), using a range of music types including classical, jazz, and sedative (Chan et al., 2010; Chen et al., 2014; Shum et al., 2014), as well as sounds including white (Afshar et al., 2016) and pink noise (Zhou et al., 2012). The effect of music on specific sleep stages, however, is less well-known with relatively few studies to date using objective sleep quality measures (Cordi et al., 2019).

Sleep consists of rapid eye-movement (REM) and non-rapid eye-movement (NREM) components, with NREM comprising various stages including slow-wave sleep (SWS) (Rechtschaffen and Kales, 1968), which is dominated by slow-wave activity (SWA) (0.5–4 Hz) consisting of delta and slow waves (<2 Hz) (Dijk et al., 1993). To date, music interventions have increased the amount of SWS (Chen et al., 2014; Cordi et al., 2019) and REM sleep (Chang et al., 2012), without changing delta power during SWS (Lazic and Ogilvie, 2007). To our knowledge, Gao et al. (2020) are the first to explore the impact of brain-wave music created from EEG during REM sleep.

Gao et al. (2020) explored the impact of SWS music (*n* = 11), REM sleep music (*n* = 13), and white noise (*n* = 9) played for 20 min before bedtime for 6 days on objective measures of sleep quality, including spectral power. The brain-wave music was created using the amplitude, period and average power of each sleep stage and translated into music pitch, duration, volume, and timbre using power law. Using this method, they aimed to compare the effects of each type of music on sleep quality and neural activation. The key finding was that after SWS brain-wave music, delta power significantly reduced, which the authors interpreted as a positive effect on sleep quality.

This is an important area of research, but in this case we offer an alternative interpretation of the results. The authors argue that lower delta power is indicative of improved sleep and SWS, citing previous research (Svetnik et al., 2017) and interpret the reduction in delta power as a reduced homeostatic pressure for delta power, as found in older adults (Landolt et al., 1996; Landolt and Borbély, 2001). However, Svetnik et al. (2017) actually found a reduction in delta power in younger adults (the age group in this study) was associated with insomnia, i.e., poorer sleep quality. Furthermore, while reduced delta activity could indicate a reduction in homeostatic sleep pressure, this is unlikely to be the case given that sleep latency after SWS music was significantly lower, and sleep efficiency somewhat higher, which are both associated with increased homeostatic sleep pressure (Dijk et al., 2010; Dijk and Landolt, 2019), while the duration of SWS was unchanged. These results suggest that the reduction of delta power is instead indicative of an impairment. This view results from both the positive consequences of increasing delta power and the negative consequences of prolonged delta power reduction (both clinically and cognitively), which is why research has focused on increasing delta power for optimal sleep quality (Marshall et al., 2006; Santiago et al., 2019).

SWS is the sleep stage most commonly associated with sleep quality due to its restorative nature (Åkerstedt et al., 1997; Dijk, 2009). To infer cause and effect, studies have focused on (a) enhancing SWS using stimulation, resulting in improvements to learning and health outcomes (Besedovsky et al., 2017; Johnson and Durrant, 2018); and (b) suppressing SWS, increasing the risk of type 2 diabetes through impaired insulin and glucose (Tasali et al., 2008; Herzog et al., 2013) and negatively affecting cognitive performance (Ferrara et al., 2000). These results collectively suggest that there are benefits to increasing delta power, whilst reducing delta power is problematic for a range of outcomes.

The brain-wave music intervention therefore appears to be unsuccessful and we suggest there are three possible reasons for this. Successful interventions use entrainment (Marshall et al., 2006; Ngo et al., 2013a) which may not be present in the current study. Auditory closed-loop stimulation entrains the sounds to the up-states of the slow-oscillations, with the slope of each individual participants slow-oscillation being measured, which then increases the amplitudes and power (Ngo et al., 2013a). The SWS brain-wave music was not, however, entrained to the SWA of each individual. Related to that, the music stimuli appeared to have no clear metrical structure and the tempo was nominally set to 120 bpm for both conditions with no consistent beat present in reality, in spite of previous findings connecting EEG periodicity to tempo (Fujioka et al., 2012). More generally, the particular mapping of EEG to music parameters is highly questionable, with no inherent relationship between average spectral power and musical timbre, for example. Finally, the stimulation was performed for only 20 min prior to sleep, when successful interventions performed prior to sleep/during sleep onset have been performed for a longer duration, e.g., 90 min (Ngo et al., 2013b). Similarly, performing auditory stimulation during SWS is more successful for improving SWA than prior to sleep/sleep onset (Ngo et al., 2013a,b). We therefore suggest that lack of entrainment, arbitrary EEG-music mapping and unfortunate stimulus timing may have contributed to the lack of success of the intervention.

In conclusion, we believe that this research does not show a positive effect of SWS brain-wave music on sleep quality; quite the contrary. We agree, however, that it is an important question and using music with characteristics specific to individual sleep stages is a positive development. The implementation in this case, however, seems to be flawed and as such has led to inconclusive findings. Future research should, therefore, focus on improving the implementation, incorporating entrainment, appropriate stimulus timing, and a music-EEG mapping grounded in existing evidence from cognitive neuroscience.

## Author Contributions

JJ and SD wrote the paper. Both authors contributed to the article and approved the submitted version.

## Conflict of Interest

The authors declare that the research was conducted in the absence of any commercial or financial relationships that could be construed as a potential conflict of interest.
